# Randomised study of systematic lymphadenectomy in patients with epithelial ovarian cancer macroscopically confined to the pelvis

**DOI:** 10.1038/sj.bjc.6603323

**Published:** 2006-08-29

**Authors:** A Maggioni, P Benedetti Panici, T Dell'Anna, F Landoni, A Lissoni, A Pellegrino, R S Rossi, S Chiari, E Campagnutta, S Greggi, R Angioli, N Manci, M Calcagno, G Scambia, R Fossati, I Floriani, V Torri, R Grassi, C Mangioni

**Affiliations:** 1 Istituto Europeo di Oncologia, Milan, Italy; 2 Università ‘La Sapienza’, Rome, Italy; 3 S. Gerardo Hospital, Monza, Italy; 4 Centro di Riferimento Oncologico, Aviano, Italy; 5 Istituto Nazionale Tumori, Fondazione G. Pascale, Naples, Italy; 6 Università ‘Campus Biomedico’, Rome, Italy; 7 Università Cattolica del ‘Sacro Cuore’, Rome, Italy; 8 Laboratory of Clinical Cancer Research, Mario Negri Institute, Milan, Italy; 9 Treviglio Hospital, Treviglio, Italy

**Keywords:** lymphadenectomy, ovarian carcinoma, surgery, randomised clinical trial

## Abstract

No randomised trials have addressed the value of systematic aortic and pelvic lymphadenectomy (SL) in ovarian cancer macroscopically confined to the pelvis. This study was conducted to investigate the role of SL compared with lymph nodes sampling (CONTROL) in the management of early stage ovarian cancer. A total of 268 eligible patients with macroscopically intrapelvic ovarian carcinoma were randomised to SL (*N*=138) or CONTROL (*N*=130). The primary objective was to compare the proportion of patients with retroperitoneal nodal involvement between the two groups. Median operating time was longer and more patients required blood transfusions in the SL arm than the CONTROL arm (240 *vs* 150 min, *P*<0.001, and 36 *vs* 22%, *P*=0.012, respectively). More patients in the SL group had positive nodes at histologic examination than patients on CONTROL (9 *vs* 22%, *P*=0.007). Postoperative chemotherapy was delivered in 66% and 51% of patients with negative nodes on CONTROL and SL, respectively (*P*=0.03). At a median follow-up of 87.8 months, the adjusted risks for progression (hazard ratio [HR]=0.72, 95%CI=0.46–1.21, *P*=0.16) and death (HR=0.85, 95%CI=0.49–1.47, *P*=0.56) were lower, but not statistically significant, in the SL than the CONTROL arm. Five-year progression-free survival was 71.3 and 78.3% (difference=7.0%, 95% CI=–3.4–14.3%) and 5-year overall survival was 81.3 and 84.2% (difference=2.9%, 95% CI=−7.0–9.2%) respectively for CONTROL and SL. SL detects a higher proportion of patients with metastatic lymph nodes. This trial may have lacked power to exclude clinically important effects of SL on progression free and overall survival.

Epithelial ovarian cancer represents a significant health problem in western countries, ranking fifth in cancer incidence and fourth in site-specific causes of cancer deaths for females, with a life time risk of about 2% (Parkin *et al*, 1988; Jemal *et al*, 2005). Ovarian cancer is potentially curable by surgery; the cure rate is, however, poor because in most patients the disease is diagnosed at an advanced stage when overall five-year survival is only about 30% (Jemal *et al*, 2005). Although there is currently no effective screening strategy for ovarian cancer, proteomics and assessment of CA-125 kinetics might make the diagnosis of early stage ovarian cancer more frequent for the future (Liotta *et al*, 2001; Skates *et al*, 2003). While the surgical procedures and the requirements for optimal intraperitoneal surgical staging of cancers apparently confined to the pelvis (International Federation of Gynecologic and Obstetrics (FIGO) stage I and II disease) (FIGO Committee on Gynecologic Oncology, 2000) are well established, the surgical approach to retroperitoneal nodes is controversial. Even when the tumour is seemingly limited to the gonads or with pelvic extension only the spread to retroperitoneal nodes is not uncommon. Involvement of pelvic nodes have been reported to occur in 8– 15% (Piver *et al*, 1978; Burghadt *et al*, 1991) and of paraaortic nodes in 5–24% of patients with stage I disease (Musumeci *et al*, 1977; Burghadt *et al*, 1991). Lymphatic spread in early ovarian cancer is a predictor of outcome with potential clinical value (Baiocchi *et al*, 1998; Di Re and Baiocchi, 2000). In fact, the presence of node metastases upstages the patients to FIGO stage IIIc disease and these patients are appropriate candidates for adjuvant postoperative chemotherapic treatments. As prevalence of lymph node metastases depends closely on the number of lymph nodes removed and examined (Carnino *et al*, 1997) we have designed a randomised trial to evaluate the role of systematic lymphadenectomy at primary surgery in patients with ovarian cancer macroscopically confined to the pelvis. Objectives of this study were to provide insight into the biologic significance of node metastases on clinical behavior and to assess the impact of systematic lymphadenectomy on progression-free and overall survival. Assessment of complications related to systematic lymphadenectomy in this group of patients was also a main objective of this study.

## PATIENTS AND METHODS

### Eligibility

Patients with histologically proven epithelial ovarian carcinoma, macroscopically confined to the pelvis and optimally debulked (residual tumour ⩽1 cm) were eligible for the study. Further eligibility criteria included age < 75 years, Karnofsky performance status ⩾80 and no previous chemo or radiotherapy treatment. The study protocol was revised and accepted by local ethical committees; informed consent was obtained from all patients in accordance with local and national legislation.

### Type of surgery

Primary surgery was aimed at removing the primary tumour and metastatic pelvic implants and included total abdominal hysterectomy, bilateral salpingo-oophorectomy, total omentectomy, appendectomy, random peritoneal biopsy specimens, peritoneal washing and the removal of all macroscopic intra-pelvic tumour.

#### Control arm

Patients enrolled in the control arm were supposed to undergo random removal of pelvic and aortic nodes (sampling) at the end of primary surgery.

#### Lymphadenectomy arm

Primary surgery as detailed above was followed by systematic pelvic and aortic lymphadenectomy.

Pelvic lymphadenectomy: The dissection begun at the origin of the external iliac vessels and continued caudally around them along the medial border of the psoas muscle; the lower limit of external iliac lymphadenectomy was represented by the deep inferior epigastric vessels. The lateral bounderies of dissection was superficially delineated by the fascia covering the psoas muscle and deeply by the fascia covering the internal obturator and levator ani muscles, the medial margin of the lymphadenectomy, was represented by an imaginary plane which is parallel to the umbelical artery and is delineated by the ombelico-pubic fascia, the bladder and the rectum.

In addition lymphatic tissue should be cleared from the obturator fossa. The clearing of the obturator fossa begun with the mobilizations of superficial obturator nodes which were removed en bloc with the lymphatic fatty tissue which has been previously separated from the internal iliac vessels to the origin of the internal pudendal vessels. Unilateral lymphadenectomy was allowed in unilateral tumours. Bilateral pelvic lymph node dissection was deemed satisfactory when at least 20 nodes were removed.

Aortic lymphadenectomy: Nodal dissection started at aortic bifurcation by removing the superficial intercavoaortic, precaval and preaortic nodal groups. Then, lymph nodes located lateral to the cava (paracaval) were separated from the vena cava, the renal capsule and the psoas muscle and removed en bloc. Afterward, displacing the vena cava and the aorta laterally and medially, the lymph nodes behind the cava (retrocaval nodal group) and the lumbar vessels (deep intercavo-aortic nodes) were separated from the prevertebral fascia and then removed. Removal of the most cranial nodes, both behind and under the left renal vein was performed after entering the right plane of dissection between the Toldt's and Gerota's fasciae, mobilising the descending colon from the renal capsule, the psoas, the ovarian pedicle and the ureter and displacing it medially.

Aortic lymph node dissection was regarded as satisfactory when at least 15 nodes were removed.

### Surgical pathology

This protocol did not recommend specific methods for processing lymph nodes but pathologists were asked to obtain two slides per node.

### First- and- second-line chemotherapy

After primary surgery and pathologic staging , patients with FIGO stage IIb-c, IIIa and IIIc for nodal involment were to receive platinum-based chemotherapy, regardless of the randomisation arm. When the study was launched the role of post-operative chemotherapy in stage I and IIa disease was controversial and the use of chemotherapy rested on the discretion of the individual investigator.

### Statistical methods

In 1990 a comprehensive program to evaluate the impact of lymphadenectomy in ovarian carcinoma was designed. Such program aimed at evaluating staging accuracy in macroscopically intrapelvic disease and survival in FIGO stage III disease (Bendetti Panici *et al*, 2005). A third trial was launched to assess the influence of lymphadenectomy performed at II look surgery after primary chemotherapy on survival. In 1991 a network of Italian hospitals coordinated at the Mario Negri Institute (Milan) started randomisation of patients with macroscopically intrapelvic ovarian cancer while in 1994 a second network of centres from Italy and other four countries started accrual of advanced ovarian cancer patients.

Randomisation with equal probability of assignment to each treatment (no-lymphadenectomy *vs* systematic lymphadenectomy) was carried out by a block arrangement balancing the treatment assignment within centre. Randomisation was performed centrally by telephone in six sites. To optimise intraoperative randomisation procedures, one site, enrolling patients also for the trial on advanced disease, randomised patients using a ‘blind envelope’ technique, that is , sealed envelopes that contained the treatment assignment. Randomisation codes were generated at the Mario Negri Institute, and the patient's envelope (identified by a registration number) was opened only after patient was enrolled.

Randomisation was carried out intraoperatively at the end of endoperitoneal surgical procedures.

Pretreatment data and operative details were collected soon after surgery. Chemotherapy and initial follow-up data were collected 6 months later and further follow-up data were collected annually thereafter. All data were sent to the Coordinating Center at the Mario Negri Institute.

The primary end point of this study was the prevalence of patients with positive retroperitoneal nodes. Lymph node involvement varies according to stage and depends closely on the number of lymph nodes removed and examined. In series of stage I patients undergoing lymph nodes sampling the prevalence ranged from 2% (Tsuruchi *et al*, 1993) to 4,2% (Carnino *et al*, 1997) while ranged from 13% (Di Re *et al*, 1989) to 24% (Burghadt *et al*, 1991) in series of reported stage I patients undergoing pelvic alone or pelvic and aortic lymphadenectomy. Therefore, the trial was planned to recruit 280 patients to demonstrate a 10% difference in the prevalence of lymph node positivity from 5% in nonlymphadenectomy arm to 15% in lymphadenectomy arm with at least 80% power (using a two-sided test and alpha of 5%). A target sample size which was suitable to detect even a rather strong effect of systematic lymphadenectomy on survival (e.g.: hazard ratio for death=0.66, which translates into increases in 5-year survival, from 80 to 86%) was deemed practically unattainable (521 patients).

Secondary endpoints were overall survival, which was defined as the time interval between the date of randomisation and the date of death from any cause, progression free survival (defined as the time from randomisation to the earliest occurrence of progression or death from any cause) and surgical morbidity.

The data from all eligible randomised patients were analyzed for survival on an intention-to-treat basis, the survival curves were estimated by the Kaplan–Meier method and compared by using the log-rank test (Kaplan and Meier, 1958). We used the Kaplan–Meier estimates of overall or progression-free survival in the control group (no lymphadenectomy) at specific time points and the hazard ratio to calculate absolute benefits at those time points according to the formula: absolute benefit=exp {HR*log[control survival]}-control survival (Parmar and Machin, 1995). Although this approach implicitly assumes proportional hazards, it is preferable to comparing differences between Kaplan–Meier curves at individual time points since is less susceptible to fluctuating results. Additional analyses were done with the Cox proportional hazards model while adjusting for multiple baseline characteristics. When adjusting for the use of postoperative chemotherapy we considered all the patients who had ad least one course of chemotherapy and we modified the model to include postoperative chemotherapy as a time-dependent covariate. The time *t* at which the value of postoperative chemotherapy covariate switched from no-chemotherapy to yes-chemotherapy was the date of the end of therapy.

Comparisons of proportions between the two groups were done by use of a two-sided *χ*^*2*^ test or a two-sided Fisher's exact test if the number of patients in a given category was five or fewer. The two-sided Kruskal–Wallis (non-parametric) test was used to compare the treatment effects of continuous variables that were expressed as median with interquartile ranges.

## RESULTS

### Accrual

**Figure 1:**
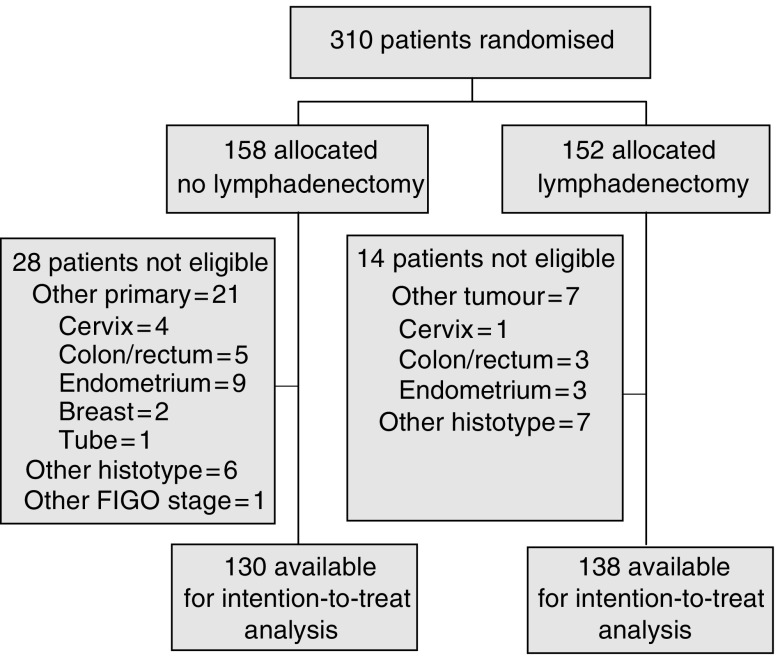
CONSORT trial flow diagram for patients with early stage ovarian cancer who were accrued into the trial.

Between January 1991 and May 2003, 310 patients were enrolled onto this study by seven participating centers. After pathology and clinical review, 42 patients were deemed ineligible. Reasons for ineligibility are detailed in Figure 1. Although there were three times as many patients with other primary in the no-lymphadenectomy group than in the lymphadenectomy group (21 *vs* 7 cases, respectively) we do not think this was a detection bias due to the different surgery conducted.

### Characteristics of patients

The characteristics of eligible patients are listed by randomised arm in Table 1 and appear well balanced across the treatment groups; of note, about 70% of patient population had FIGO stage I at random and only nine out of the 72 patients with stage II disease had residual tumour (⩽1 cm).

### Perioperative evaluation

In Table 2 we reported the median and 25th–75th percentiles of resected nodes by treatment arm. Forty-four patients in the lymphadenectomy arm were stage Ia and underwent ipsilateral lymphadenectomy (median of resected nodes in this subgroup=36; pelvic 19, aortic 17).

As a consequence of the high number of resected nodes in the lymphadenectomy arm, a higher proportion of these patients had positive nodes at histologic examination than patients randomised to control (overall: 22 *vs* 9%, *P*=0.007; stage I at random: 18 *vs* 4%; stage II at random: 31 *vs* 20%). As expected, nodal involvement was correlated with tumour grade (in the lymphadenectomy arm, 31% of patients with grade III tumour had metastatic nodes *vs* 11% of patients with grade I/II tumour; *P*=0.004) and histological type of tumour (in the lymphadenectomy arm, 33% of patients with serous or undifferentiated tumour had metastatic nodes *vs* 10% of patients with other cell types; *P*=0.005). Among the patients with nodal metastases in the lymphadenectomy arm, 21% had pelvic, 54% aortic and 25% pelvic plus aortic involvement. Table 3 shows that systematic lymphadenectomy had a significant impact on surgical parameters such as median operative times, blood loss, and proportion of patients undergoing blood transfusions. Median hospital stay for patients undergoing systematic lymphadenectomy was one day longer than patients undergoing no-lymphadenectomy (*P*=0.003). Neither the number of intraoperative nor perioperative/late complications was statistically different between the two groups (eight cases *vs* four and eight cases *vs* 16 in the control and lymphadenectomy arm, respectively). Most of the difference in late morbidity was due to formation of lymphocysts and lymphedema, which occurred in eight cases in the lymphadenectomy group *vs* none in the control arm. Adhesive small bowel obstruction occurred in one patient after lymph nodes sampling only and in two patients after lymphadenectomy. There were no surgery related deaths.

### Post-operative adjuvant chemotherapies

Patients (61%) received adjuvant chemotherapy after surgery (66 and 56% for control and systematic lymphadenectomy, respectively; *P*=0.11). Patients (90%) with positive nodes and 56% of patients with negative nodes received postoperative chemotherapy; a statistically significant difference in the use of chemotherapy emerged between groups in node negative patients (66 and 51% of patients on control arm and systematic lymphadenectomy arm, respectively, *P*=0.03). No differences in chemotherapy schedules emerged between groups. 97% of patients who received postoperative chemotherapy underwent platinum based mono- (51%) or multi- (46%) agent regimens while 3% patients received non platinum-based chemotherapy regimens.

### Progression-free and overall survival

**Figure 2:**
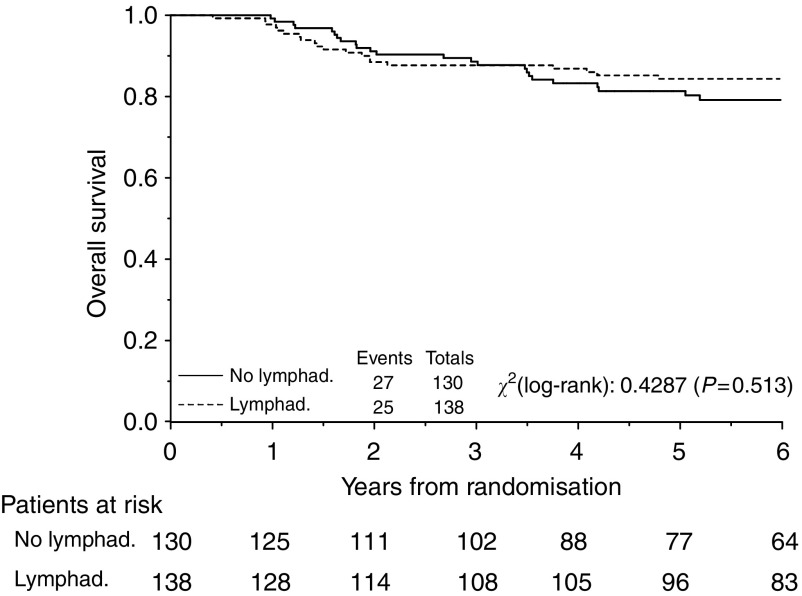
Overall survival (OS) for patients with optimally debulked early ovarian carcinoma undergoing systematic aortic and pelvic lymphadenectomy (Lymphad.) *vs* lymph nodes sampling only (No lymphad). Five-year overall survival was 81.6 and 84.0% (difference=2.4%, 95% CI=−8.3 to 8.9%) respectively for lymph nodes sampling only and lymphadenectomy.

**Figure 3:**
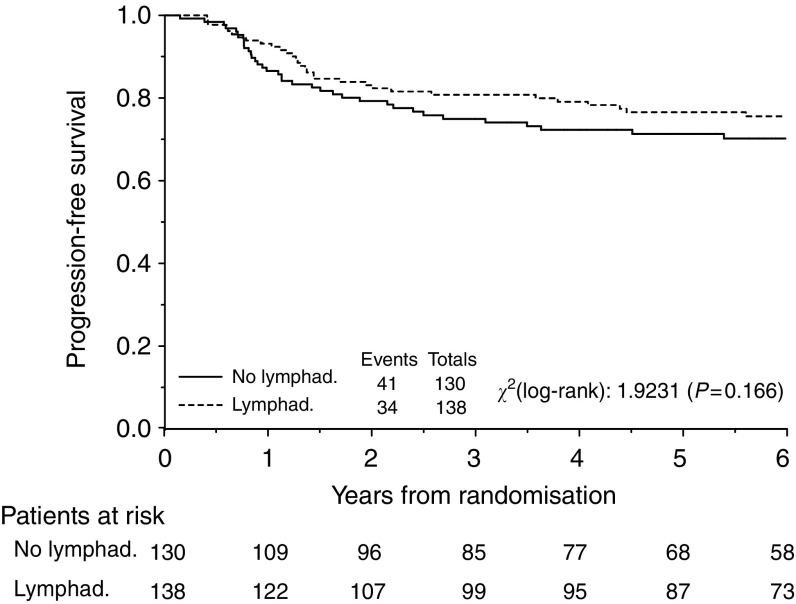
Progression-free survival (PFS) for patients with optimally debulked early ovarian carcinoma undergoing systematic aortic and pelvic lymphadenectomy (Lymphad.) *vs* lymph nodes sampling only (No lymphad.). Five-year progression-free survival was 73.4 and 78.3% (difference=4.9%, 95% CI=−5.9 to 12.5%) respectively, for lymph nodes sampling only and lymphadenectomy.

At a median follow-up of 87.8 months (25th–75th percentiles: 62.7 to 120.6 months) tumour has recurred in 69 patients (25.7%) and 52 patients (19.4%) have so far died, six without evidence of disease (2.2%). Recurrence was experienced by 30% of patients of the control arm and by 22% of patients who underwent systematic lymphadenectomy and the pattern of disease recurrences is shown in Table 4. Figures 2 and 3 depict the overall and progression-free survival, respectively, for all eligible patients.

Comparison of the Kaplan–Meier curves for progression free survival gave a hazard ratio of 0.73 (95% CI=0.46– 1.14; *P*=0.17), which translates into absolute increases in 3- and 5-year progression-free survival of 6.2% (95% CI=−3.0 to 12.6%) and 7.0% (95% CI=−3.4 to 14.3%), respectively. Median progression-free survival was not reached for patients in either arms.

Comparison of the Kaplan–Meier curves for overall survival gave a hazard ratio of 0.83 (95% CI=0.48–1.44), which translates into absolute increases in 3- and 5-year overall survival of 1.8% (95% CI=−4.6– 5.7%) and 2.9% (95% CI=−7.0–9.2%), respectively. Median overall survival was still non reached in both groups.

A Cox proportional hazards analysis was performed to adjust the treatment comparison for baseline characteristics. When histologic grade was taken into account the new hazard ratios for death and progression remained virtually unchanged (Table 5). Even when we took into account the unbalance in postoperative chemotherapy use between treatment arms the hazard ratios for death and progression were very similar (HR=0.85, 95% CI=0.49 to 1.48, *P*=0.56 and HR=0.72, 95% CI=0.45 to 1.14, *P*=0.16 respectively).

Finally we tried to evaluate the prognostic value of positive aortic and/or pelvic lymph nodes inside each treatment group. Metastatic nodal involvement was a strong negative prognostic factor in the control arm (HR=4.61, CI=1.94– 11.0, *P*=0.0005) while nodal involvement was no longer a determinant of survival in the lymphadenectomy arm (HR=0.71, CI=0.24–2.06, *P*=0.52).

## DISCUSSION

To our knowledge, this is the first randomised trial of patients with ovarian carcinoma macroscopically confined to the pelvis that has compared systematic lymphadenectomy and lymph nodes sampling only. The main result of this study is that a statistically significant higher proportion of patients was found to have metastatic involvement of pelvic and/or aortic nodes in the lymphadenectomy group than in the control group (22 *vs* 9%, respectively, *P*=0.007). That means an upstaging of these apparent early stage ovarian cancers to a stage IIIc disease. Although systematic lymphadenectomy was associated with an improvement in progression-free and overall survival this improvement was not statistically significant.

Systematic lympadenectomy is a major surgical major procedure but this study provided evidence that it was feasible in the framework of a pragmatic multicentre randomised trial. In the lymphadenectomy arm the median number of removed pelvic and aortic lymph nodes was 24 and 21, respectively. Overall, the median number of removed nodes was 47 in the lymphadenectomy arm and 5.5 in the control arm. The incidence of postoperative complications was similar between the groups but the median operating time in the lymphadenectomy arm was about 90 min longer and the blood loss about 300 ml higher than in the control arm, and 14% more patients underwent a blood transfusion in the experimental group. The median postoperative hospital stay was one day longer in the lymphadenectomy group than in the control arm. Considering that endoperitoneal surgery for early ovarian cancer is relatively simple the extra burden of the systematic lymphadenectomy seems accetable in terms of operation length and complications.

The prevalence of lymph node involvement depends closely on the stage of the disease and the number of lymph nodes removed and examined (Burghadt *et al*, 1991; Carnino *et al*, 1997). Our study shows that when lymphadenectomy is systematically performed, nearly a fourth of patients with ‘early-stage’ ovarian cancer turns out to have retroperitoneal lymphatic spread. This is still a conservative estimate of the true prevalence of nodal involvement as only two sections were examined for each node and therefore several micrometastastic deposits (0.2 cm or smaller) and also some macrometastases might have been missed. In early stage ovarian cancer, positive nodes can be found in nine to 25% of patients (Baiocchi *et al*, 1998). Such variabilility is due to the relatively small size of patient series and the heterogeneity of techiques used to detect and remove retroperitoneal nodes which span from simple sampling (Chen and Lee, 1983; Lanza *et al*, 1988; Carnino *et al*, 1997) to systematic lymphadenectomy (Di Re *et al*, 1989; Burghadt *et al*, 1991; Benedetti Panici *et al*, 1993; Carnino *et al*, 1997; Baiocchi *et al*, 1998). Our findings are in keeping with these data but represent the first direct comparison of the two surgical approaches to retroperitoneal nodes and are not hampered by the methodologic constrains of retrospective analyses. Our study showed that, in the lymphadenectomy arm, nodal status correlated with grading and histologic subtype. Information about grading and histology are usually obtained from biopsies well in advance of primary surgery so that clinicians can optimise the lymphadenectomy diagnostic efficacy the moment they plan it in patients with serous/undifferentiated or grade 3 tumours.

Nodal metastases have been shown to independently correlate with survival in epithealial ovarian cancer, both in advanced (Burghadt *et al*, 1991; Carnino *et al*, 1997) and early stage (Baiocchi *et al*, 1998). Univariate analysis revealed that the presence of metastatic nodes correlated with survival (HR=4.61) in the control group. Conversely, in the lymphadenectomy group, nodal involvement was no longer a prognosticator for survival suggesting that the removal of all nodes increased the likelihood of complete tumour debulking. Bendetti Panici *et al* (2005) showed that in patients with advanced ovarian cancer undergoing systematic lymphadenectomy node positivity was still correlated with survival as although systematic lymphadenectomy did not contribute to optimal tumour debulking and therefore to clinical outcome.

Standard of postoperative care for patients with FIGO stage IIb to IV ovarian cancer is adjuvant chemotherapy. On the contrary, before the publication of ICON1-Action studies (International, 2003) there was no compelling evidence to support early institution of cytotoxic chemotherapy in stage I to IIa disease. Therefore, in such cases, the use of postoperative chemotherapy was left at the discretion of the treating physician who tried to identify a subgroup of patients at high risk for relapse depending on features that are predictive of inferior survival like incomplete staging and higher stage or tumour grading. Nowadays the majority of patients with early stage ovarian cancer will require adjuvant platinum-based with or without taxanes chemotherapy. As a significant proportion of patients undergoing systemic lymphadenectomy were upstaged to stage IIIc that warrants the institution of postoperative chemotherapy we would have expected more patients treated with chemotherapy in the lymphadenectomy arm than in the control arm. On the contrary, due to the lack of evidence based guidelines, it seemed that clinicians preferred to err on the side of overtreatment in node negative patients who entered the control arm. Thus, the control arm was associated with a trend toward higher use of postoperative chemotherapy. In particular, there were seven patients out postoperative chemotherapy in the control arm. The Action trial (Trimbos *et al*, 2003) suggested that the patients with early-stage ovarian cancer after optimal surgical staging (and nodal sampling was still considered an adequate approach to retroperitoneum) showed neither an overall survival nor a relapse free survival benefit from adjuvant chemotherapy. Our study showed that 22% of apparent early-stage ovarian cancer harbour more advanced disease and indirectly reinforce a conservative approach aimed at sparing low-risk patients with negative nodes (after lymphadenectomy) unnecessary cytotoxic treatments.

There were no clear differences in the pattern of relapses and, specifically, there was a similar number of retroperitoneal relapses between the two arms.

This study was underpowered to detect a limited but still clinically important effect of systematic lymphadenectomy on progression-free or overall survival. Although nonstatistically significant, the punctual estimates of the HRs for progression or death from any cause favored lymphadenctomy and this finding is even more interesting considering that less patients received postoperative chemotherapy in the lymphadenectomy arm than in the control arm. In conclusion, systematic lymphadenectomy seems a relatively safe and acceptable surgical procedure when performed in selected gynecologic oncology institutions and it guarantees the optimal accuracy of staging which in turn allows to tailor postoperative treatments.

## Figures and Tables

**Table 1:** Clinical and tumour characteristics by treatment arm

	**No lymphadenectomy (130 patients)**		**Lymphadenectomy (138 patients)**	
	***n***	**%**	***n***	**%**
Median age (25th–75th percentiles)	52 (44–59)		51 (43–60)	
				
*FIGO Stage (at random, before pathologic staging)*				
I	90	69.2	102	73.9
II	39	30.0	33	23.9
				
Missing data	1	0.8	3	2.2
				
*Residual tumour*				
None	126	96.9	133	96.4
⩽1 cm	4	3.1	5	3.6
				
*Tumour grade*				
1	20	15.4	30	21.7
2	41	31.5	29	21.0
3	65	50.0	72	52.2
				
Missing data	4	3.1	7	5.1
				
*Cell type*				
Serous	43	33.1	61	44.2
Endometriod	34	26.2	24	17.4
Mucinous	22	16.9	14	10.1
Clear-cell	19	14.6	16	11.6
Undifferentiated	8	6.1	7	5.1
Other	2	1.5	8	5.8
				
Missing data	2	1.5	8	5.8

**Table 2:** Number of resected nodes by treatment arm

	**No lymph. (130 patients)**	**Lymph. (138 patients)**	
	**Median no. (25th–75th percentiles)**	**Median no. (25th–75th percentiles)**	***P***
Pelvic nodes	3.5 (0–8.5)	24 (15–33)	<.0001
Aortic nodes	1 (0–4)	21 (15–30)	<.0001
Pelvic and aortic nodes	5.5 (0–12)	47 (33–63)	<.0001
Missing data	2	8	

**Table 3:** Operative details and postoperative hospital stay

**Surgical outcome**	**No lymph. (130 patients)**	**Lymph. (138 patients)**	***P***
Median operating time (min) (25th–75th percentiles)	150 (120–180)	240 (210–300)	<.0001
Missing data	6	17	
			
Median blood loss (ml) (25th–75th percentiles)	300 (200–550)	600 (400–900)	<.0001
Missing data	9	18	
			
Patients transfused (%)	21.85	35.5	0.012
			
Median hospital stay (days) (25th–75th percentiles)	6 (5–7)	7 (6–9)	0.003
Missing data	9	14	

**Table 4:** Site of disease recurrence by treatment arm

	**No lymph. (130 patients)**		**Lymph. (138 patients)**	
	**no.**	**%**	**no.**	**%**
No recurrence	91	70.0	108	78.0
				
*Recurrence*	39	30.0	30	22.0
Pelvic	13	9.2	11	8.0
Intraperitoneal	8	6.1	5	3.6
Retroperitoneal	4	3.1	2	1.4
Distant site	0	0	3	2.2
Multiple	13	10.0	9	6.5
Missing data	1	0.7	0	0

**Table 5:** Multivariable cox proportional hazards analysis for progression-free and overall survival

	**Progression-free survival**		**Overall survival**	
	**HR (95%CI)**	***P***	**HR (95%CI)**	***P***
*Treatment arm*				
No lymphadenectomy^a^		0.16		0.56
Lymphadenectomy	0.72 (0.46–1.14)		0.85 (0.49–1.47)	
				
*Grade*				
1 or 2^a^		0.01		0.08
3	1.84 (1.14–2.96)		1.66 (0.93–2.95)	

HR=hazard ratio CI=confidence interval.

a Reference category
